# Long-term results of a prospective randomized trial comparing tension-free vaginal tape versus transobturator tape in stress urinary incontinence

**DOI:** 10.1007/s00192-023-05527-z

**Published:** 2023-04-19

**Authors:** Heini Salo, Henri Sova, Johanna Laru, Anne Talvensaari-Mattila, Virva Nyyssönen, Markku Santala, Terhi Piltonen, Sari Koivurova, Henna-Riikka Rossi

**Affiliations:** Department of Obstetrics and Gynecology, PEDEGO Research Unit, Medical Research Center, Oulu University Hospital, University of Oulu, Kajaanintie 50, Box 5000, 90014 Oulu, Finland

**Keywords:** Stress urinary incontinence, Mid urethral sling, Tension-free vaginal tape, Transobturator tape

## Abstract

**Introduction and hypothesis:**

This study was aimed at investigating the long-term effectiveness of minimally invasive mid-urethral sling (MUS) surgery and at comparing the outcomes between retropubic (tension-free vaginal tape, TVT) and transobturator tape (TOT) methods in the treatment of stress urinary incontinence (SUI) and mixed urinary incontinence (MUI) with a predominant stress component in a long-term follow-up of a randomized controlled trial.

**Methods:**

This work is a long-term follow-up study of a previous prospective randomized trial conducted in the Department of Obstetrics and Gynecology at Oulu University Hospital between January 2004 and November 2006. The original 100 patients were randomized into the TVT (*n*=50) or TOT (*n*=50) group. The median follow-up time was 16 years, and the subjective outcomes were evaluated using internationally standardized and validated questionnaires.

**Results:**

Long-term follow-up data were obtained from 34 TVT patients and 38 TOT patients. At 16 years after MUS surgery, the UISS significantly decreased from a preoperative score in the TVT (11.88 vs 5.00, *p*<0.001) and TOT (11.05 vs 4.95, *p*<0.001) groups, showing a good long-term success of the MUS surgery in both groups. In comparing the TVT and TOT procedures, the subjective cure rates did not differ significantly between the study groups in long-term follow-up according to validated questionnaires.

**Conclusion:**

Midurethral sling surgery had good long-term outcomes in the treatment of SUI and MUI with a predominant stress component. The subjective outcomes of the TVT and TOT procedures were similar after a 16-year follow-up.

**Supplementary information:**

The online version contains supplementary material available at 10.1007/s00192-023-05527-z

## Introduction

Stress urinary incontinence (SUI) is defined as involuntary urine leakage during physical exertion or while sneezing or coughing, whereas urge incontinence is involuntary loss of urine associated with urgency [1]. Incontinence is defined as mixed urinary incontinence (MUI) when patients suffer from both stress and urge incontinence [1]. SUI affects up to 40–50% of the female population, and 18% of them suffer from MUI [2–5]. This constitutes a large social burden with a profound effect on women’s quality of life (QoL) [5, 6]. Furthermore, its impact on health care costs is significant [5]. The risk factors for SUI and MUI include age, parity, and obesity, and the prevalence of urine incontinence is likely to increase as the population ages and the obesity rate increases [3, 7].

The first-line treatment of urinary incontinence is conservative, and it includes lifestyle modifications (weight control, cessation of smoking, etc.) and physiotherapy. However, conservative treatment is usually inadequate. Therefore, minimally invasive mid-urethral sling (MUS) operations are currently the gold standard of surgical treatment for SUI [8–10]. In MUI, the treatment is chosen individually based on the predominant type of incontinence [6]. Tension-free vaginal tape (TVT) was first described in 1995, and it had good success rates (84–95%), with minimal invasiveness and complication rates [8, 11]. However, complications, although rare, including bladder, bowel, and major blood vessel injuries, led to the development of the TOT technique in 2001. TOT procedures use two techniques: outside–in (TOT) or inside–out (TVT-O) techniques [8, 11]. Several studies have proven no significant difference between the objective and subjective outcomes of the TVT and TOT tape (TOT and TVT-O) techniques in short-term (1–5 years) follow-up studies [4, 12, 13]. There are fewer studies on long-term follow-up, most of which have demonstrated comparable success rates for MUS procedures [4, 5, 10, 14]. However, some studies have suggested that TVT could have better outcomes than TOT [2, 9, 15]. As the aim of MUS surgery is to reduce incontinence and thus improve subjective QoL, validated questionnaires have been widely applied as a measure of the effectiveness of the surgery. The most commonly used questionnaires are the Urinary Incontinence Severity Score (UISS), Pelvic Floor Distress Inventory (PFDI-20), and Patient Global Index of Improvement (PGI-I) [16–18].

The aims of this study were to investigate the effectiveness of minimally invasive MUS surgery at an average of 16 years’ follow-up, and to compare the subjective outcomes of TVT and TOT methods in the treatment of SUI and MUI in the long-term follow-up of a randomized controlled trial (RCT).

## Materials and methods

This work is a 16-year follow-up study evaluating the long-term benefits of MUS surgery compared with a previous prospective randomized controlled study on 100 women suffering from SUI or MUI with a predominant stress component. The original study protocol was approved by the regional ethics committee and the present study has approval of the hospital district of Northern Ostrobothnia, Finland (124/2020). All the women underwent surgery at the Department of Obstetrics and Gynecology at Oulu University Hospital between January 2004 and November 2006 [8]. In the original study, the patients were randomized with sealed and numbered envelopes into a TVT (*n*=50) or a TOT (*n*=50) procedure. Power calculation was not performed in the original RCT and therefore we calculated the post hoc power by assuming a non-inferior study with 85% success rate in both groups, a non-inferiority margin of 5% and with α=0.05, giving a post hoc power of 17%. The procedures were performed by a single experienced surgeon (MS) with equal experience in TVT and TOT, and standard knitted monofilament polypropylene tapes were used for both techniques: Gynecare® tape for TVT surgery and Monarc® tape for TOT surgery.

Preoperative urinary incontinence was evaluated with two internationally standardized and validated questionnaires: the UISS and Detrusor Instability Score (DIS) [16, 19]. The severity of incontinence was assessed using the UISS. This questionnaire consists of 10 fundamental urinary incontinence symptom impact items that are scored from 0 to 2 based on the severity of the symptom. Questionnaire items include questions considering impact on daily activities, work life, sexual life, pad usage and psychological wellbeing. The maximum score of the UISS questionnaire is 20, and a score of more than 8 points was considered severe incontinence. The degree of symptoms of an overactive bladder was estimated using the DIS questionnaire, which ranged from a score of 0 to 20, with ≤7 points indicating stress incontinence and >7 points indicating urge or mixed incontinence [19]. To evaluate the immediate success of the operation, we collected the UISS and DIS scores 3 months after the operation from the hospital patient records.

For this long-term follow-up study, we attempted to recruit all patients from the original prospective randomized study. Of the 100 patients, 3 were deceased during follow-up (3 in the TVT group and 0 in the TOT group). Patients were contacted by postal letter, or by phone if there was no response to the letter. Long-term follow-up data were collected using the same internationally standardized and validated questionnaires (UISS and DIS) as preoperatively. The effect of surgery on well-being was measured with the PGI-I questionnaire [17], which had a scale of 1–7, with 1 corresponding to “much better” and 7 to “much worse” compared with the preoperative situation [17]. The effects of surgery were dichotomized into poor (PGI-I 1–4) and good (PGI-I 5–7) long-term outcomes. Pelvic floor symptoms were estimated using the PFDI-20 [18], with a maximum score of 100. UISS, DIS, PGI-I and PFDI-20 questionnaires were sent by postal mail; if no answer was received by letter, an interview was conducted by telephone.

The IBM SPSS Statistics version 28 was used for the statistical analysis. Continuous variables were calculated as means or medians with standard deviations or interquartile range, and categorical variables were calculated as frequencies. The differences in continuous variables were analyzed using an independent samples *t* test or a Mann–Whitney *U* test, as appropriate, and a paired samples *t* test and Chi-squared test were used to analyze the differences in the categorical parameters. A two-sided *p* value <0.05 was considered statistically significant.

## Results

**Fig. 1:**
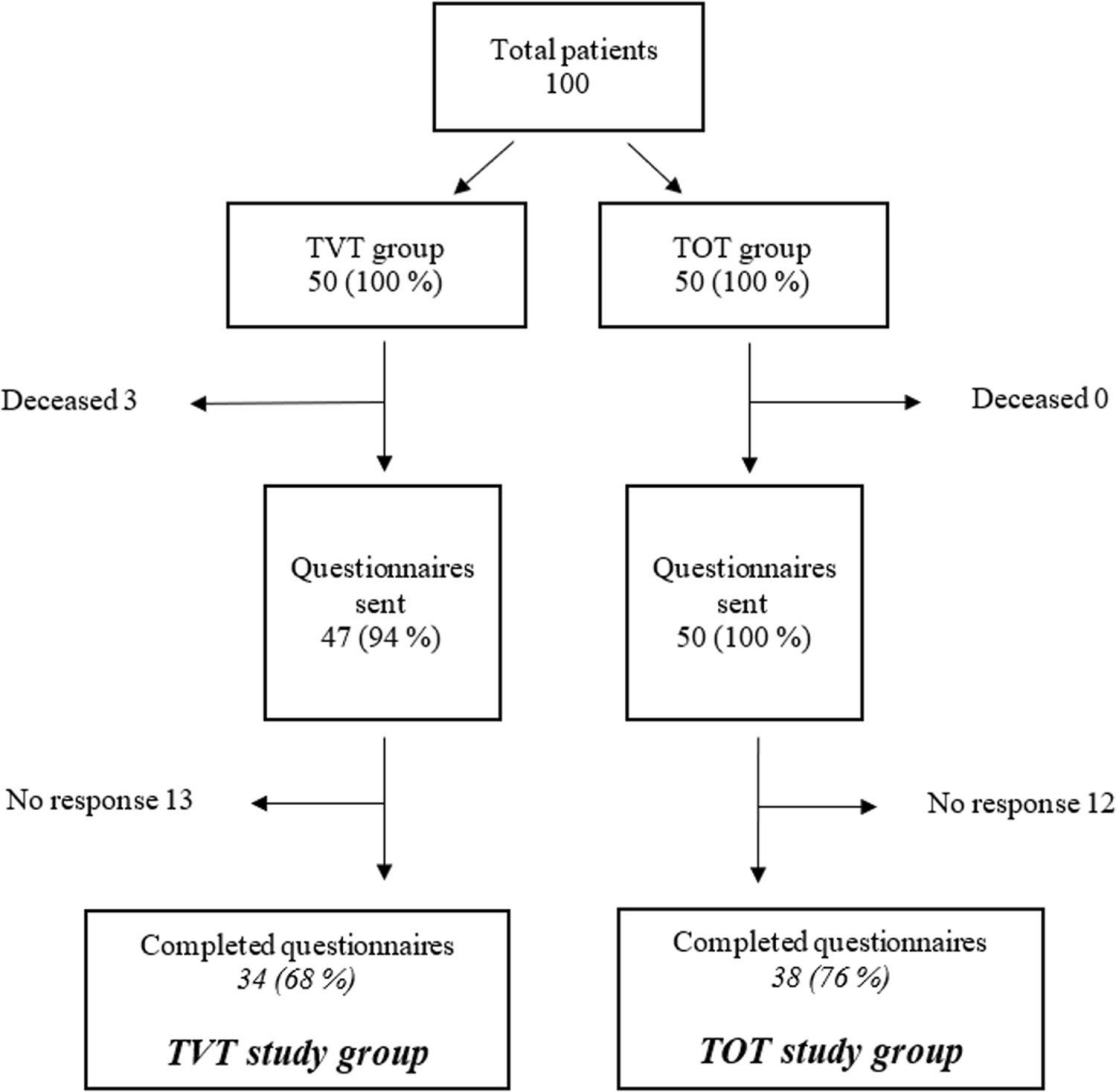
Flowchart and description of the study population, *n* (%). *TOT* transobturator tape, *TVT* tension-free vaginal tape

Responses and consent were received from 72 patients; among them, 34 underwent TVT, and 38 underwent TOT (Fig. 1). Twenty-five patients did not respond. The nonresponders were older at age 73.88±10.81 years vs 69.36±7.53 years (*p*<0.001), and their initial body mass index (BMI) was lower than that of the ones who remained in the follow-up (26.84±4.22 vs 27.04±3.86, *p*<0.001). Moreover, preoperative UISS was higher in the nonrespondent group than in the respondent group (12.08±3.87 vs 11.44±3.43, *p*<0.001), and preoperative DIS was lower in the nonrespondent group than in the respondent group (6.36±2.77 vs 6.45±2.85, *p*<0.001). Furthermore, nonresponders’ UISS (3.20±4.32 vs 1.24±2.33, *p*<0.001) and DIS (4.68±2.95 vs 4.03±2.66, *p*<0.001) at 3 months after the operation was higher (Supplemental Table 1a). When comparing TVT and TOT groups within the nonrespondents, there were no significant differences in preoperative characteristics or at 3 months UISS or DIS scores (Supplemental Table 1b).

The preoperative patient characteristics are shown in Table 1. The median follow-up time was 15.94 (± 0.78) years in the TVT group and 16.11 (± 0.80) years in the TOT group. The mean BMI before surgery was slightly higher in the TOT group than in the TVT group (27.95±3.79 vs 26.06±3.76, *p*=0.039). No significant difference was found in the mean age of the patients or preoperative UISS or DIS between the study groups. Prior hysterectomy had been performed on 9 patients (26.5%) in the TVT group and 11 patients (28.9%) in the TOT group (*p*=0.815). Prior colporrhaphy was performed on 3 (8.8%) patients in the TVT group and 3 (7.9%) patients in the TOT group (*p*=0.887). MUS surgery had not been performed previously on any of the patients. One patient (2.9%) had undergone a prior Burch operation in the TVT group and 3 (7.9%) in the TOT group (*p*=0.360; Table 1).

**Table 1:** Preoperative patient characteristics

Characteristic	TVT (*n*=34)	TOT (*n*=38)	*p* value
Preoperative BMI (kg/m^2^)	26.06±3.76	27.95±3.79	**0.039**
Age at operation (years)	50.94±7.65	53.58±7.10	0.081
Preoperative UISS	11.88±3.54	11.05±3.35	0.482
Preoperative DIS	7.03±2.98	5.95±2.67	0.469
Prior hysterectomy	9 (26.5%)	11 (28.9%)	0.815
Prior colporrhaphy	3 (8.8%)	3 (7.9%)	0.887
Prior Burch	1 (2.9%)	3 (7.9%)	0.360

Data are shown as mean (standard deviation) or numbers (percentages)

Significance tests for continuous variables were performed by using the independent samples *t* test or the Mann–Whitney *U* test, as appropriate

*p* value < 0.05 was considered significant

*TVT* tension-free vaginal tape, *TOT* transobturator tape, *BMI* Body Mass Index; *UISS* Urinary Incontinence Severity Score, *DIS* Detrusor Instability Score

Statistically significant values (*p* < 0.05) are presented in bold

**Fig. 2:**
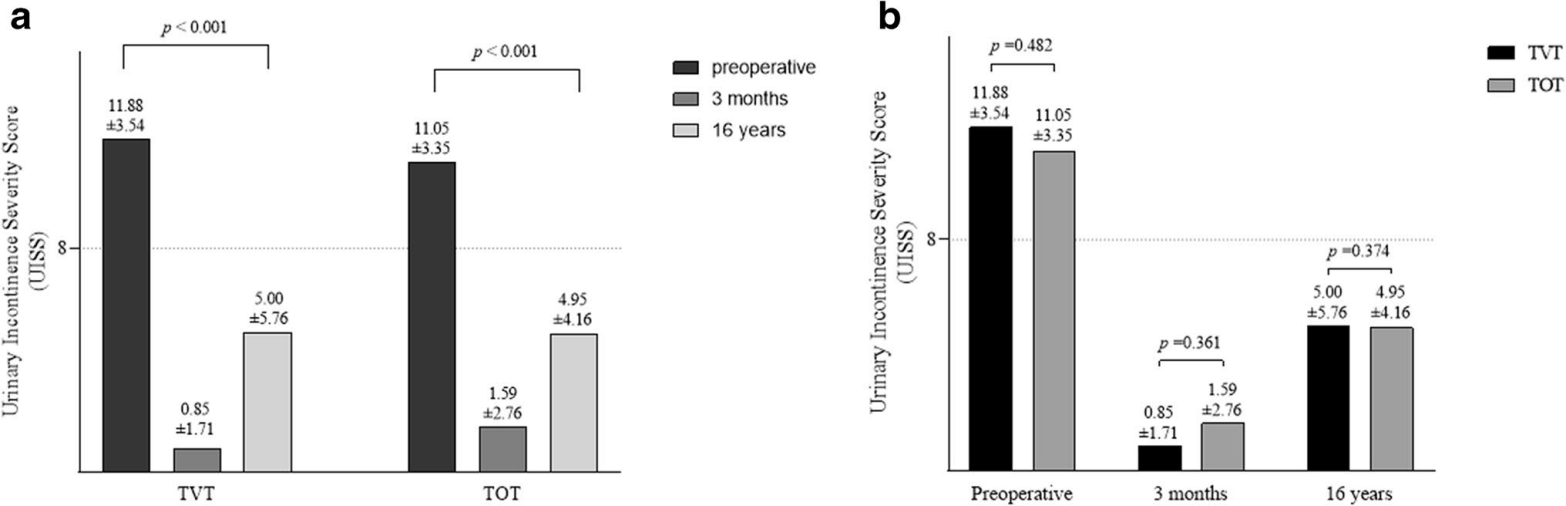
**a** The effectiveness of tension-free vaginal tape (*TVT*) and transobturator tape (*TOT*) based on Urinary Incontinence Severity Score (*UISS*) preoperatively, at 3 months, and at 16 years after the operation. *p* values according to paired samples *t* test. **b** Comparison of UISS preoperatively, at 3 months, and at 16 years after the operation between TVT and TOT groups. More than 8 points in the UISS is considered as severe incontinence (*dashed line*)

In the 16-year follow-up, the UISS increased in both study groups compared with the 3-month score, but the scores were still significantly lower than the preoperative scores, showing the good long-term success of both procedures (Fig. 2a). When comparing TVT and TOT, the preoperative UISS of the study groups was comparable (TVT: 11.88±3.54 vs TOT: 11.05±3.35, *p*=0.482). In the 3-month follow-up, the there was no difference in the UISS between the TVT group and the TOT group (UISS 0.85±1.71 vs 1.59±2.76, *p*=0.361). In the 16-year follow-up, the UISS was similar in the TVT group and the TOT group (5.00±5.76 vs 4.95±4.16, *p*=0.374; Fig. 2b).

**Fig. 3:**
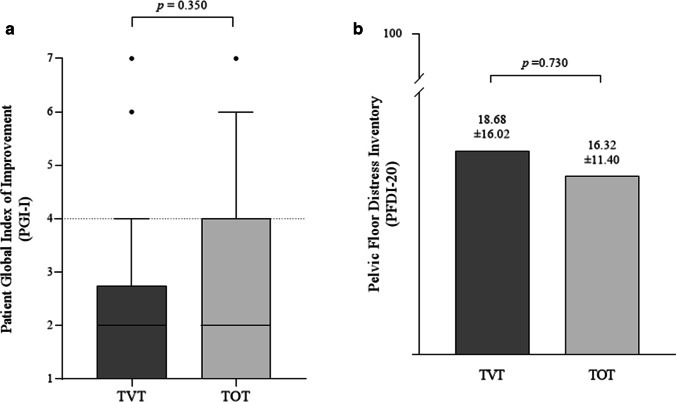
**a** Patient Global Index of Improvement (*PGI-I*) at 16 years after the operation. Box-plot whiskers stand for 10th percentile and 90th percentile of the data set. **b** Pelvic Floor Distress Inventory (PFDI-20) at 16 years after the operation. *p* values according to independent-samples *t* test. *TOT* transobturator tape, *TVT* tension-free vaginal tape

No significant difference was found in the PGI-I score (2.0±2 in TVT vs 2.0±3 in TOT, *p*=0.417) between the study groups 16 years after surgery (Fig. 3a). When the PGI-I score was dichotomized into good and poor outcomes, there were almost twice the number of patients in the TOT group reporting poor outcomes compared with the TVT group (11 [28.9%] vs 5 [15.6%], *p*=0.186), although the difference was not statistically significant. The variability in PGI-I was higher in the TOT group than in the TVT group. The women who reported poor long-term outcomes after MUS surgery were older (55.44±8.13 years vs 51.26±6.92 years, *p*=0.045) and had a higher preoperative BMI (28.76±4.24 vs 26.49±3.70, *p*=0.042) than the cases in the good outcome group.

The PFDI-20 scores were equal in the TVT group and the TOT group (18.68±16.02 vs 16.32±11.40, *p*=0.730; Fig. 3b). Similarly, DIS was comparable in the groups, both in the 3-month follow-up (4.09±2.77 in TVT vs 3.97±2.59 in TOT, *p*=0.974) and the 16-year follow-up (6.66±3.96 in TVT vs 6.11±2.99 in TOT, *p*=0.665).

## Discussion

This long-term follow-up study of a prospective RCT showed that MUS surgery, as a treatment for SUI and MUI with a predominant stress component, has a good long-term subjective outcome. Similar subjective outcomes were found for the TVT and TOT procedures. To the best of our knowledge, this is the first long-term follow-up study using a previous RCT study population with comparable study groups and to standardize the factors related to the success of the operation.

This study showed good subjective outcomes after MUS surgery in a long-term follow-up. Preoperatively, most of our patients suffered from severe incontinence (UISS>8). According to the 3-month follow-up, the UISS was significantly lower than the preoperative score, showing good primary success of the MUS procedures. Even after the 16-year follow-up, the UISS remained at a significantly lower level than the preoperative score, showing good long-term success of MUS. Furthermore, no significant difference was found in the PGI-I score between the TVT and TOT groups 16 years after surgery. Dichotomizing the PGI-I score into good (1–3) and poor (4–7) outcomes, there were slightly more patients in the TOT group reporting poor outcomes than in the TVT group, although the difference was not statistically significant. However, the patients with poor subjective outcomes according to the PGI-I score had a higher age and BMI, which could explain the slight difference between the TVT and TOT groups. Our findings are comparable with those of previous studies, which showed that MUS surgery has good long-term effectiveness in the treatment of SUI [4, 20, 21]. A recent study by Braga et al. showed subjective and objective cure rates of 88.8% and 91.7% respectively for a TVT operation with a 20-year follow-up [22]. Similar results were found by Bakas et al., who analyzed patients after a TVT operation with a 17-year follow-up and reported objective and subjective cure rates of 83.9% and 78.6% respectively [23]. However, only a few publications match the long follow-up time of our study, and these publications have been generally conducted with smaller study populations than ours. Moreover, none of these publications used a randomized operation technique between the TVT and TOT groups. The mild increase in the symptoms of incontinence observed in our study after the 16-year follow-up could be derived from multiple causes, such as aging and lifestyle factors. It is possible that the result of MUS surgery could have somewhat deteriorated in the long term, but according to our results, the outcome of incontinence was still significantly better than in the preoperative situation.

To our knowledge, there are only a limited number of studies comparing the outcomes of retropubic and transobturator MUS techniques. The present study showed no difference in the subjective outcomes between the TVT and TOT procedures in a long-term follow-up when the confounding factors related to operation success, such as the patients’ characteristics and the surgeons’ experience, were considered. We found that in a 16-year follow-up, the severity of urinary incontinence, pelvic floor function, and postoperative improvement according to validated questionnaire scores were similar in the TVT and TOT groups. These results are in accordance with those of several other studies demonstrating equal long-term effectiveness between TVT and TOT procedures [10, 12, 14]. A large systematic review and meta-analysis by Leone Roberti Maggiore et al. [5] and a Cochrane database review by Ford et al. [4] showed no difference in the long-term subjective cure rates between TVT and TOT. Controversially, a recent Finnish register-based study by Tulokas et al. [2] suggested that TVT might have better long-term efficacy than TOT in the treatment of SUI (risk for re-operation for SUI 2.6% after TVT vs 10.6% after TOT, OR=3.6). In their study, the assessment of effectiveness was based on the re-operation data obtained from the hospital patient register. However, confounding factors, such as patients’ BMI and age or surgeon’s learning curve, which are known to be related to the outcome of surgery, were not considered [24]. Moreover, the number of patients in the study groups differed significantly (3,280 vs 245). The higher number of patients in the TVT group indicated that surgeons could have more experience with the TVT method than with the less commonly used TOT method, which could have caused bias. In a recent RCT by Offiah et al., TVT was found to be significantly superior to TOT in treating SUI (41.8% in the TVT group vs 21.8% in the TOT group) in a 12-year follow-up after surgery [15]. In this study, the difference in the improvement of symptoms (estimated using the PGI-I) between the groups favored the TVT procedure (79.7% vs 77.2%). Unlike the present study, there were multiple surgeons in their study. In a multicenter, randomized trial TOMUS by Richter et al., TVT had a higher treatment success rate than TOT, both objectively (80.8% vs 77.7%) and subjectively (62.2% vs 55.8%) in a 1-year follow-up [25]. Furthermore, a 5-year follow-up study to the TOMUS study was conducted, in which TVT had a higher treatment success rate than TOT (51.3% vs 43.4%) [26]. However, patients’ subjective satisfaction with the TVT and TOT procedures was similar (79% vs 85%). Also, in a systematic review and meta-analysis by Tommaselli et al., TOT was associated with an almost twofold lower subjective cure rate than TVT [9]. However, this study involved unselected patients and nonrandomized trials. Altogether, the discrepancy in the previous studies could be due to the heterogeneity of the patient populations, the variability of operation methods and surgeon’s experiences, the differences in study designs, and the lengths of the follow-up times.

The TVT procedure was the first minimally invasive MUS operation to be used effectively to treat SUI. The TVT route can expose patients to rare but, nonetheless, severe complications (bladder and bowel perforation, severe bleeding), which can be avoided using the TOT route [4, 11]. Furthermore, TVT tapes may cause more postoperative voiding difficulties and postoperative de novo urgency than TOT tapes [27]. In terms of de novo urgency, we found an equivalent postoperative DIS showing an equal rate of overactive bladder symptoms between the TVT and TOT groups in a 16-year follow-up. Thus, our results are in accordance with those of previous studies [9, 15], in which the incidence of de novo urgency was approximately 7–10% for both techniques. The data on incontinence recurrence or the need for repeat incontinence surgery after TVT and TOT techniques are controversial [4], and we were not able to assess them in the present data set.

Long-term follow-up with a high response rate (72%) and a prospective randomized controlled design are the strengths of this study. This study is the first long-term study to be based on a previous RCT with minimal differences in the baseline characteristics (i.e., a slightly higher BMI in the TOT group). Validated questionnaires were used to assess the subjective cure, which is the main focus of gynecological incontinence surgery. Furthermore, the procedures were performed by one experienced surgeon, reducing the bias of a learning curve effect on the clinical outcome. This study also has some limitations. The number of patients in the study was quite small, and the limited number of cases could underestimate the statistically significant differences between the study groups. Furthermore, despite the high response rate, missing data may lead to a slight bias, whereas nonresponders were older, they had more severe incontinence and the immediate success of MUS was lower, which may have had a slight effect on the evaluation of the long-term outcome. Although we were able to analyze the subjective cure rate using a validated questionnaire, the lack of assessment of postoperative pain and an objective cure rate can be considered limitations. Moreover, the PFDI-20 questionnaire is aimed at investigating pelvic floor problems, not at purely assessing difficulties in urinary incontinence. We did not have complete information about the patients’ general state of health, and the subjective symptom assessment could not have been completely reliable in all study cases, especially among the elderly and/or patients with memory disorder. Although we were able to standardize the experience of the surgeon, it makes the results less generalizable, and it is possible that the one surgeon favored or had more expertise in one method than in the other. Last, our data were limited in evaluating the need for re-operation and the number of complications related to different surgical methods.

Midurethral sling surgery is a minimally invasive and effective treatment for SUI when conservative treatment does not provide sufficient symptom relief. Initially, the focus was on the TVT method, but complications after TVT led to the development of TOT methods. However, owing to a lack of evidence for their effectiveness and long-term follow-up data, there has been uncertainty about the superiority of one of these two methods over the other. Our prospective follow-up of an RCT shows that TVT and TOT procedures are equally effective in terms of the subjective cure rate in a long-term follow-up when the factors related to MUS surgery success are standardized. Despite the limited sample of women presented in this long-term follow up, this study provides insightful data that could also be useful in future meta-analyses. Further studies are needed in terms of complications and need for reoperations related to different MUS techniques. Considering these results, we recommend choosing the method in accordance with the surgeon’s best experience.

## Supplementary information

ESM 1 (DOCX 17 kb)
