# Supplementary material for: Long-term results of a prospective randomized trial comparing tension-free vaginal tape versus transobturator tape in stress urinary incontinence
Source: Int Urogynecol J. 2023 Apr 19;34(9):2249–56. doi: 10.1007/s00192-023-05527-z (PMC10506929; doi:10.1007/s00192-023-05527-z)
Supplement: Supplementary file 1 — (DOCX 17 kb) [file 192_2023_5527_MOESM1_ESM.docx]

**Supplemental tables**

**Supplemental table 1a**. *Characteristics of non-respondent and respondents*

| Characteristic | Non-respondent (*n*=25) | respondent group (*n*=72) | *p* value |
| --- | --- | --- | --- |
| Preoperative BMI (kg/m²) | 26.84 ± 4.22 | 27.04 ± 3.86 | < 0.001 |
| Age at operation  (years) | 73.88 ± 10.81 | 69.36 ± 7.53 | < 0.001 |
| Preoperative UISS | 12.08 ± 3.87 | 11.44 ± 3.43 | < 0.001 |
| Preoperative DIS | 6.36 ± 2.77 | 6.45 ± 2.85 | < 0.001 |
| UISS at 3 months | 3.20 ± 4.32 | 1.24 ± 2.33 | < 0.001 |
| DIS at 3 months | 4.68 ± 2.95 | 4.03 ± 2.66 | < 0.001 |

*Data are shown as mean (standard deviation).*

*Significance tests for continuous variables were performed by using the independent samples t test or the Mann–Whitney U test, as appropriate. P value <.05 was considered significant.*

*Abbreviations: BMI:Body Mass Index; UISS:Urinary Incontinence Severity Score; DIS:Detrusor Instability Score.*

**Supplemental table 1b.** *Characteristics of non-respondents in each study group*

| Characteristic | TVT (*n*=13) | TOT (*n*=12) | *p* value |
| --- | --- | --- | --- |
| Preoperative BMI (kg/m²) | 27.08 ±4.77 | 26.58 ±3.73 | 0.777 |
| Age at operation (years) | 54.69 ±9.85 | 58.83 ±11.39 | 0.340 |
| Preoperative UISS | 11.85 ±5.00 | 12.33 ±2.31 | 0.761 |
| Preoperative DIS | 6.31 ±3.50 | 6.42 ±1.83 | 0.924 |
| UISS at 3 months | 3.46 ± 4.37 | 2.92 ± 4.44 | 0.983 |
| DIS at 3 months | 4.69 ± 2.81 | 4.67 ± 3.23 | 0.380 |

*Data are shown as mean (standard deviation).*

*Significance tests for continuous variables were performed by using the independent samples t test or the Mann–Whitney U test, as appropriate. P value <.05 was considered significant.*

*Abbreviations: BMI:Body Mass Index; UISS:Urinary Incontinence Severity Score; DIS:Detrusor Instability Score.*
